# Universal power-law of terahertz optical properties of borosilicate, tellurite, and chalcogenide glass families

**DOI:** 10.1038/s41598-023-29345-x

**Published:** 2023-02-08

**Authors:** Nicholas J. Tostanoski, S. K. Sundaram

**Affiliations:** Terahertz Waves Science and Technology Laboratory (T-Lab), Inamori School of Engineering, The New York State College of Ceramics, Alfred University, Alfred, NY 14802 USA

**Keywords:** Materials for optics, Materials science

## Abstract

Terahertz (THz) time-domain spectroscopy (TDS, THz-TDS) was used to measure THz optical properties, i.e., refractive indices and absorption coefficients, of borosilicate, tellurite, and chalcogenide glass families. We observe that the THz optical properties depend on glass compositions. THz refractive indices recorded an increasing trend from borosilicate to chalcogenide and to tellurite glass families. Our results demonstrate the ability to select a glass family, system, and composition to target THz optical properties for potential use in THz optical and photonic applications. We report K and β fitting parameters for the power-law model used to describe these properties and show how it can be universally applied to several glass families.

## Introduction

Glasses can be utilized as various passive as well as active optical components, e.g., waveguides, windows, lenses, etc., at THz frequencies due to the ability to tailor, customize, and control optical properties including low or high refractive index, reduced dispersion, and absorption coefficient, that can be selected for any desired applications. Knowledge of THz refractive indices and absorption coefficients across the THz bandwidth for numerous glass families strengthens the possibilities for glasses to be used in this frequency range for various applications encompassing broad areas of THz optics and photonics, specifically focused on communication systems^1^, security and defense^2^, and medical diagnosis^3^.

THz applications include quality control and examination in various fields including industrial food production, transportation of packaged materials, inspection of artwork, inspection and examination of semiconductor wafers, moisture analysis for agriculture, and in the paper, automotive, and pharmaceutical industries^4–8^. THz reflection imaging is used in biomedical diagnosis of diseases as THz radiation has limited penetration in living tissue and can be used for near surface identification of cancerous tissues, e.g., skin and breast cancer, due to the unique THz signature^1,3,9^. For example, the pharmaceutical industry utilizes THz spectroscopy for a variety of specialized uses including analytical characterization, material identification, and to study drug delivery systems. It has specifically been used to determine the degree of crystallinity, coating thickness, uniformity, roughness, porosity, and defects, e.g., cracks and delamination, seen on tablet coatings^10–13^.

Naftaly et al.^14–16^, Kang et al.^17^, and Ravagli et al.^18^ have reported THz optical and dielectric properties of select commercially available silicate glasses including, polycrystalline fused quartz, amorphous fused silica, and silicate glasses of B 270® (modified soda-lime crown glass), BK7® (borosilicate glass), Pyrex® (borosilicate glass), N-Zk7® (zinc crown glass), SF® series (dense flint glass), and SK10® (dense barium crown glass). Borosilicate, tellurite, and chalcogenide glasses are defined as silicate-containing oxide, non-silicate oxide, and non-oxide glass families, respectively. Different glass families have vastly different compositions, structures, e.g., structural units, connectivity, and networks, and resulting properties, e.g., THz refractive indices and absorption coefficients. Storm et al.^19,20^ and Schlomann^21^ analyzed THz absorption coefficients using a power-law model, $n\left(\nu \right)\alpha \left(\nu \right)={K\left(h\nu \right)}^{\beta }$ or the simplified form of $n\alpha ={K\times f}^{\beta }$, where $n\left(\nu \right)$ is the frequency dependent refractive index, $\alpha \left(\nu \right)$ is the frequency dependent absorption coefficient, K is determined by material properties, and β is a constant dependent on glass composition. K is defined as $K=\frac{{{e}^{*}}^{2}N{k}^{2}}{{\mathrm{\hbar }}^{2}\rho c{V}_{D}^{3}}$, where $N$ is the density of charge fluctuations of amplitude (${e}^{*}$), k is the local field correction factor (n^2^ + 2)/3, $\mathrm{\hbar }$ is the reduced Planck constant, $\rho$ is the mass density, $c$ is the speed of light in a vacuum, and V_D_ is the Debye velocity of sound. K increases approximately with the fourth power of the refractive index. Storm et al.^19,20^ reviewed the THz far infrared absorption parameters for select glasses including SiO_2_, B_2_O_3_, GeO_2_, As_2_S_3_, Se, As_2_Se_3_, among other compositions, where the β parameters were found to be ~ 2.

Naftaly et al.^14–16^ have reviewed the power-law model, determining K and β parameters for all studied glasses. The β parameter was generally found to be ~ 2, with β increasing up to ~ 2.8 as a function of glass composition and introduction of more polarizable constituents. Kang et al.^17^ and Ravagli et al.^18^ further expanded on THz optical and dielectric properties of glasses by focusing on the chalcogenide glass family in terms of the power-law and reported K and β parameters. Ge–As–S, Ge–Ga–Se, Ge–As–Ga–Se, and Ge–Sb–Se glasses had a β parameter of ~ 2, agreeing with silicate glass studies, while La–Ga–S, La–Ga–S–Se, and Ge–As–Se glasses reported a relatively high β parameter of ~ 3, attributed to larger coordination number of La.

We have performed in-depth structural studies for all three glass families, while also reporting THz refractive indices across the measured THz bandwidth, ultimately resulting in structure-THz property relationships for the borosilicate, tellurite, and chalcogenide glass families. We suggest the readers review our previous works for structure-THz property relationship, i.e., in-depth structural and THz study, of each glass family^22–24^. THz-TDS has been used to study the refractive index and absorption coefficient at THz frequencies of select compositions within various glass families and how it can be used in glass science and engineering as a nondestructive characterization technique^25^.

We extend THz optical and dielectric properties of glasses and analyze, for the first time, the results using the power-law to obtain K and β parameters to include silicate-containing oxide, non-silicate oxide, and non-oxide glass families. We present our novel THz optical properties and power-law observations and interpretations. We aim to evaluate and validate the power-law for three different glass families to determine universality of application of the law and reveal new science in terms of the validity of the universal power law.

## Experimental procedure

Borosilicate, tellurite, and chalcogenide glasses were produced, with compositions shown in Table 1, and discussed in detail elsewhere^22–24^. Structural studies have been carried out to provide a comprehensive description of each glass systems structure through Raman spectroscopy, and additional spectroscopies, seen in previous work^22–24^. The sodium borosilicate glass system was selected within the borosilicate glass family, ultimately studying two tie line, NaBSi and BNaSi, series, studying the replacement of SiO_2_ for B_2_O_3_ with constant Na_2_O at 20 mol% and SiO_2_ for Na_2_O with constant B_2_O_3_ at 20 mol%, respectively. Within the tellurite glass family, the sodium tungsten tellurite (NWT) and lanthanum tungsten tellurite (LWT) glass systems were examined. Both systems had the network modifier content, e.g., Na_2_O and La_2_O_3_, held constant while replacing one network former for another network former, e.g., TeO_2_ for WO_3_. Within the non-oxide chalcogenide glass family, the vitreous selenium (Se), arsenic sulfide (As–S), arsenic selenide (As–Se), germanium selenide (Ge–Se), and germanium arsenic selenide (Ge–As–Se) glass systems were studied.

**Table 1:** Experimental (nominal) borosilicate, tellurite, and chalcogenide glass compositions, THz refractive indices (n) and absorption coefficients (α) at 0.5 THz, and the K and β fitting parameters to power-law relation, $n\alpha ={K\times f}^{\beta }$, for all studied glasses.

Glass	Na_2_O (mol%)	B_2_O_3_ (mol%)	SiO_2_ (mol%)	La_2_O_3_ (mol%)	WO_3_ (mol%)	TeO_2_ (mol%)	Ge (at.%)	As (at.%)	S (at.%)	Se (at.%)	n	α (cm^−1^)	K (cm^−1^)	β
NaBSi1	20	10	70	–	–	–	–	–	–	–	2.59	8.71	69.6	1.55
NaBSi2	20	17.5	62.5	–	–	–	–	–	–	–	2.31	6.15	56.4	2.01
NaBSi3	20	25	55	–	–	–	–	–	–	–	2.26	4.99	47.6	2.13
NaBSi4	20	32.5	47.5	–	–	–	–	–	–	–	2.03	1.95	16.5	2.05
NaBSi5	20	40	40	–	–	–	–	–	–	–	2.10	3.92	32.9	2.02
BNaSi1	5	20	75	–	–	–	–	–	–	–	1.99	2.12	17.7	2.08
BNaSi2	10	20	70	–	–	–	–	–	–	–	2.05	2.03	16.9	2.04
BNaSi3	15	20	65	–	–	–	–	–	–	–	2.25	4.94	43.2	1.94
BNaSi4	20	20	60	–	–	–	–	–	–	–	2.36	5.66	46.9	1.76
BNaSi5	25	20	55	–	–	–	–	–	–	–	2.43	7.78	73.8	1.96
NWT1	22	–	–	–	5	73	–	–	–	–	4.46	38.98	687	1.95
NWT2	22	–	–	–	15	63	–	–	–	–	4.66	39.67	741	1.95
NWT3	22	–	–	–	25	53	–	–	–	–	4.76	44.06	715	1.74
NWT4	22	–	–	–	35	43	–	–	–	–	4.49	39.66	619	1.78
NWT5	22	–	–	–	45	33	–	–	–	–	4.43	34.09	547	1.83
NWT6	22	–	–	–	55	23	–	–	–	–	4.23	34.27	456	1.61
LWT1	–	–	–	18	15	67	–	–	–	–	4.40	31.77	455	1.70
LWT2	–	–	–	18	24	58	–	–	–	–	4.18	28.69	470	1.95
LWT3	–	–	–	18	33	49	–	–	–	–	3.83	29.98	364	1.65
LWT4	–	–	–	18	42	40	–	–	–	–	3.55	22.95	266	1.68
LWT5	–	–	–	18	51	31	–	–	–	–	4.08	37.57	444	1.54
LWT6	–	–	–	18	60	22	–	–	–	–	4.26	26.12	401	1.81
Se	–	–	–	–	–	–	–	–	–	100	2.48	11.46	37.9	0.48
As_15_S_85_	–	–	–	–	–	–	–	15	85	–	2.22	1.90	11.2	1.46
As_20_S_80_	–	–	–	–	–	–	–	20	80	–	2.33	2.00	14.0	1.40
As_25_S_75_	–	–	–	–	–	–	–	25	75	–	2.44	2.66	18.6	1.53
As_30_S_70_	–	–	–	–	–	–	–	30	70	–	2.55	3.18	23.8	1.52
As_35_S_65_	–	–	–	–	–	–	–	35	65	–	2.65	3.72	29.0	1.53
As_40_S_60_	–	–	–	–	–	–	–	40	60	–	2.82	2.93	31.2	1.87
As_10_Se_90_	–	–	–	–	–	–	–	10	–	90	2.61	3.70	28.6	1.56
As_20_Se_80_	–	–	–	–	–	–	–	20	–	80	2.78	3.97	35.1	1.65
As_30_Se_70_	–	–	–	–	–	–	–	30	–	70	2.90	5.01	47.4	1.70
As_40_Se_60_	–	–	–	–	–	–	–	40	–	60	3.13	4.97	53.6	1.77
As_50_Se_50_	–	–	–	–	–	–	–	50	–	50	2.93	4.59	38.3	1.5
As_60_Se_40_	–	–	–	–	–	–	–	60	–	40	2.73	3.65	25.4	1.35
Ge_10_Se_90_	–	–	–	–	–	–	10	–	–	90	2.61	9.01	40.2	0.65
Ge_20_Se_80_	–	–	–	–	–	–	20	–	–	80	2.69	11.81	47.2	1.00
Ge_30_Se_70_	–	–	–	–	–	–	30	–	–	70	2.72	10.09	41.2	1.19
Ge_40_Se_60_	–	–	–	–	–	–	40	–	–	60	2.90	13.64	77.8	1.34
Ge_10_As_10_Se_80_	–	–	–	–	–	–	10	10	–	80	2.71	11.58	47.4	1.19
Ge_10_As_20_Se_70_	–	–	–	–	–	–	10	20	–	70	2.90	10.53	53.1	1.33
Ge_20_As_10_Se_70_	–	–	–	–	–	–	20	10	–	70	2.84	13.16	52.3	1.38
Ge_10_As_30_Se_60_	–	–	–	–	–	–	10	30	–	60	3.01	10.81	54.3	1.30
Ge_20_As_20_Se_60_	–	–	–	–	–	–	20	20	–	60	2.82	9.58	44.6	1.43
Ge_30_As_10_Se_60_	–	–	–	–	–	–	30	10	–	60	2.73	9.88	44.4	1.63
AMTIR 1	–	–	–	–	–	–	33	12	–	55	2.87	7.74	41.8	1.22
IRG 22	–	–	–	–	–	–	33	12	–	55	2.86	4.67	31.8	1.57
IRG 24	–	–	–	–	–	–	10	40	–	50	2.89	5.89	38.2	1.19

**Figure 1:**
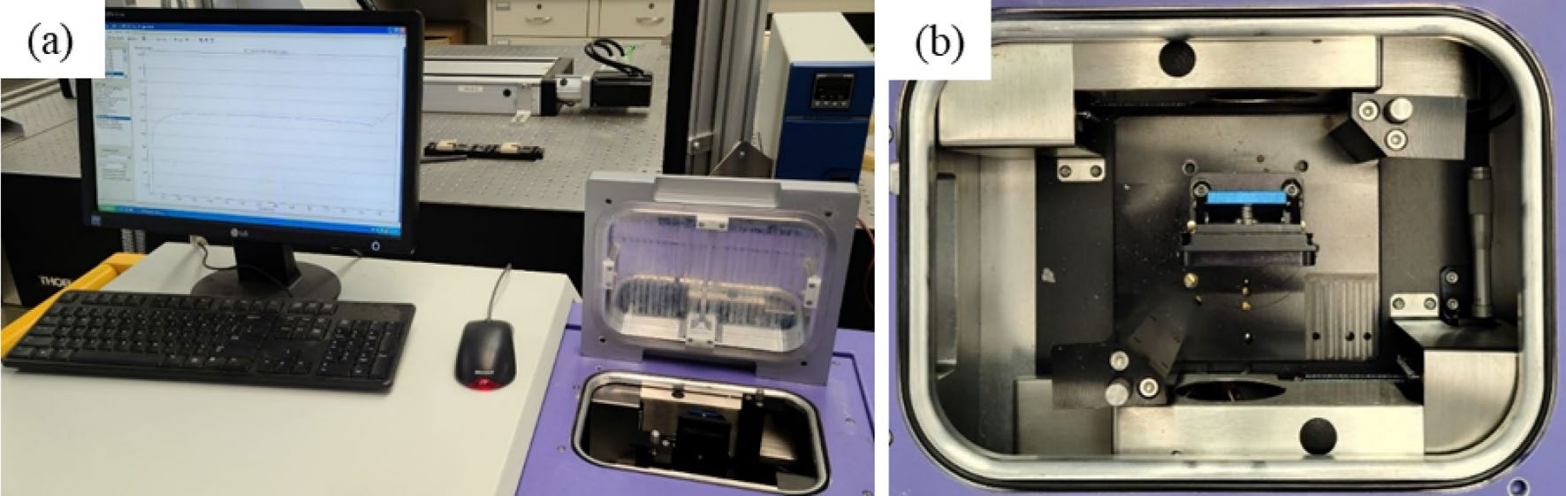
(**a**) THz-TDS experimental setup at Alfred University used for the measurements in this study and (**b**) transmission configuration sample holder, taken from our previous work^25^.

A Teraview TPS Spectra 3000 (Teraview, Cambridge, UK) was used for THz-TDS characterization. THz spectra were collected from 0.2 to 2.0 THz under ambient conditions in transmission mode, with a reference spectrum collected in pure nitrogen conditions. THz radiation was produced using a mode-locked Ti:Sapphire laser with an 800 nm central wavelength, a repetition rate of 80 MHz, and pulse duration of 100 fs. Each sample was scanned at five different locations and averaged. 3000 scans were collected for each THz spectra with a 1.2 cm^−1^ resolution. Figure 1 shows the experimental setup used for the THz-TDS measurements, as seen in our previous work^25^.

We determined K and β parameters by plotting $n\left(\nu \right)\alpha \left(\nu \right)$ as a function of frequency (THz) and then fit to the power-law function, $n\alpha ={K\times f}^{\beta }$. At a certain frequency, the transmitted beam becomes indistinguishable from the noise measured by the THz-TDS due to attenuation. Beyond this frequency, termed cutoff frequency, the low signal-to-noise ratio hinders accurate determination of material properties. We observe distinct cutoff frequencies for each glass family, influencing the measured bandwidth of THz optical properties.

## Results and discussion

**Figure 2:**
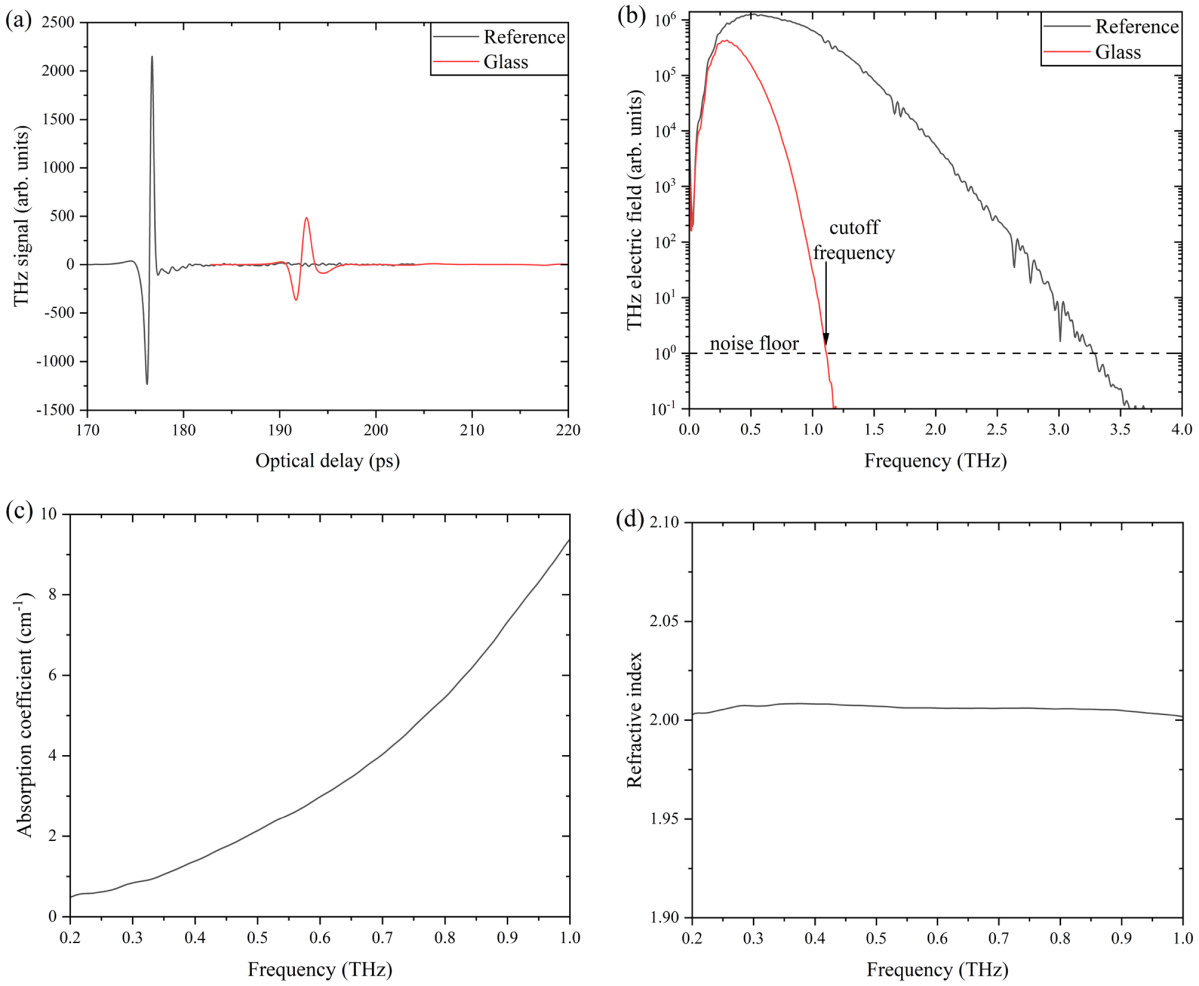
Example set of THz-TDS raw data and resulting optical properties of a glass sample. (**a**) THz time-domain waveform, (**b**) THz electric field frequency domain spectrum, and (**c**) absorption coefficient and (**d**) refractive index spectra of a glass sample at THz frequencies.

Figure 2 shows a set of experimental THz-TDS raw data and the optical properties obtained from a standard measurement for a glass sample. A reference spectrum is initially collected in pure nitrogen conditions, followed by the glass sample. The signal amplitude is reduced due to the sample absorption and the phase is shifted by the refractive index of the sample, providing compositional information. A Fourier transformation of the time-domain waveform results in the frequency domain. The cutoff frequency is obtained from the THz electric field frequency domain using the noise floor. The absorption coefficient and refractive index are obtained directly from the dataset using the amplitudes of the electric fields and relative phases of both reference and sample, respectively.

**Figure 3:**
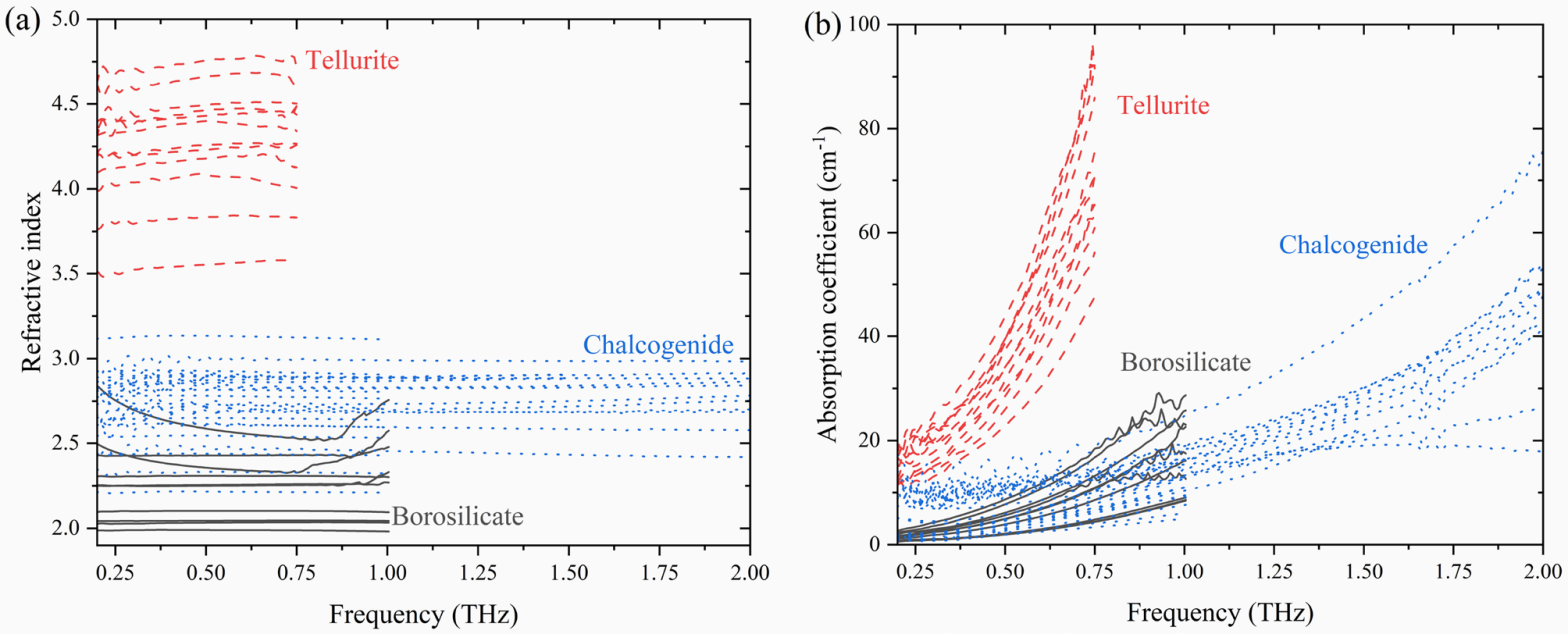
THz (**a**) refractive indices and (**b**) absorption coefficients of all glasses. Oscillations are due to the etalon effect as previously discussed.

Figure 3 shows the (a) refractive indices and (b) absorption coefficients of all the glasses studied. These properties reveal distinct trends across the three glass families. THz refractive indices and absorption coefficients measure an increase from the borosilicate to chalcogenide to tellurite glass families. This trend confirms glass families containing more polarizable constituents with increased polarizability record a larger absorption coefficient and refractive index across the THz bandwidth. Select measured glasses possess optical interference (oscillations or variability) in the THz refractive index and absorption coefficient due to the etalon effect at low THz frequencies, i.e., from 0.2 to 0.5 THz. This behavior is explained due to the sample thickness, where the reduced thickness, e.g., less than 1 mm, is comparable to the wavelength of the incident THz waves used in the THz-TDS measurement. This behavior would not be observed if the sample thickness was greater than 1 mm. We have recently studied the accuracy and reliability of our commercial THz-TDS instrument at Alfred University by performing measurements at the National Institute of Standards and Technology (NIST) using a custom THz-TDS apparatus^24^. Our study confirms that the two distinct instruments provide remarkably accurate THz-TDS optical property results and allows us to place confidence in the ability of our THz-TDS at Alfred University to measure accurate results.

The borosilicate glass family has the lowest recorded THz optical properties, as these glasses do not contain high concentration of polarizable constituents. However, we observe a rather significant range in refractive indices and absorption coefficients within the borosilicate glass system. We attribute the variability in refractive indices to the glass network and structural units. Polymerized and depolymerized borosilicate networks without and with formation of non-bridging oxygen atoms, associated with SiO_4_ silicate tetrahedra, are likely one explanation for the lower and higher refractive indices differences, respectively.

Chalcogenide glass family and numerous systems possess general increased THz optical properties, serving as the midpoint for the studied families, while also possessing an increased THz bandwidth. Specific chalcogenide glass systems, and elemental composition and constituents, allow for a wide range of THz optical properties. It is important to note there is overlap in the THz refractive indices of the chalcogenide and borosilicate glass families, suggesting the use of either glass family to achieve desired THz optical properties for potential applications. It is shown that chalcogenide glasses possess the ability to extend to higher refractive indices, than those seen in borosilicate glasses, expanding the availability of THz optical properties.

The highest THz refractive indices and absorption coefficients are measured in the tellurite glass family due to higher concentration of polarizable constituents in the form of WO_3_ and TeO_2_. There is a distinct increase in THz optical properties for tellurite glasses, while also possessing a wide range of measured properties, i.e., refractive indices from 3.5 to 4.75, when compared to borosilicate and chalcogenide glasses.

**Figure 4:**
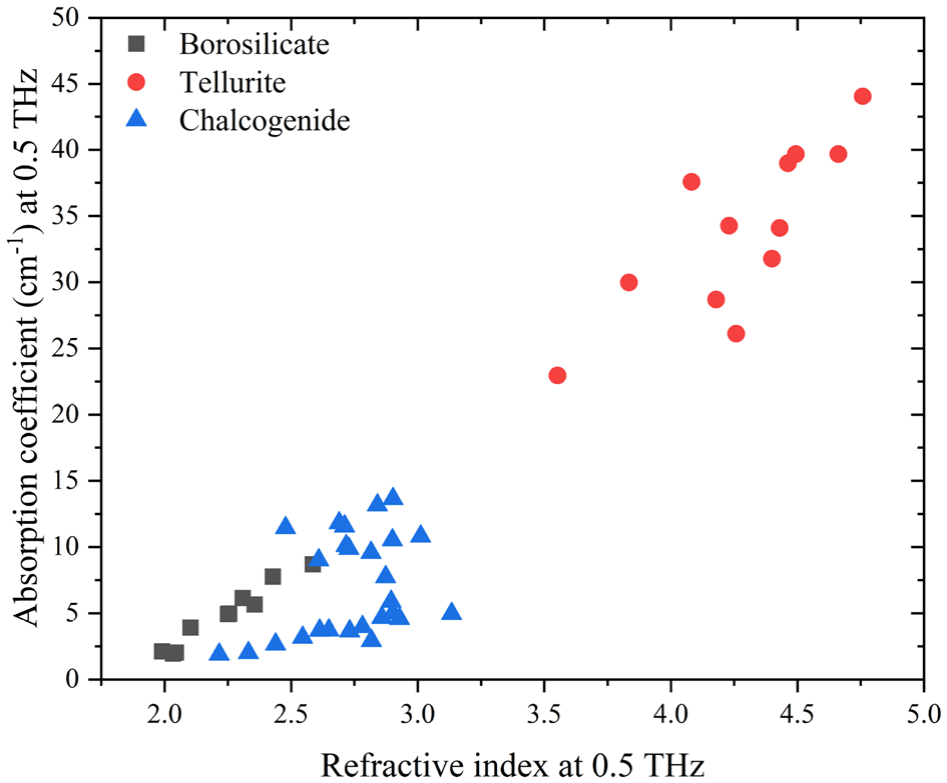
0.5 THz absorption coefficient and refractive index correlation.

Figure 4 shows the 0.5 THz absorption coefficient and refractive index correlation for all studied glasses. Broad THz optical property trends are observed within each glass family, showing the variations of THz properties that can be achieved by adjusting the composition of a glass system. Our data shows how desired THz optical properties can be targeted and achieved through selection of a glass family, system, and composition. This correlation allows for a wide selection of THz refractive index and absorption coefficient values among the three studied systems. Such a correlation is valuable for predicting and designing glass compositions for optical applications across the THz region.

**Figure 5:**
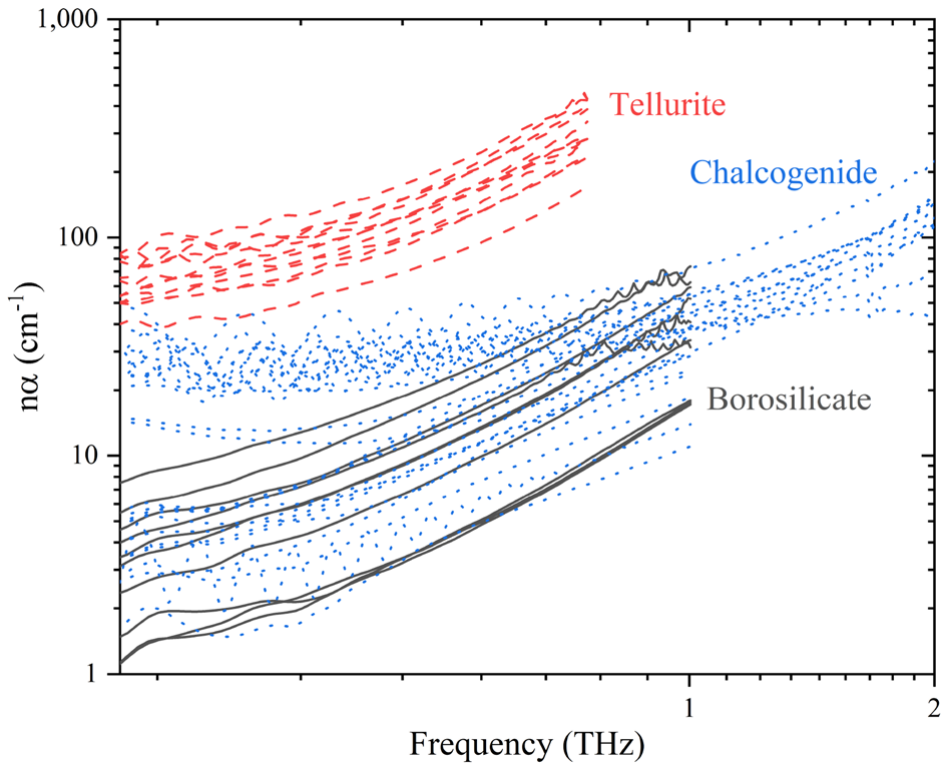
The product of the THz refractive index and absorption coefficient. Oscillations are due to the etalon effect as previously discussed.

Figure 5 shows the product of the THz refractive index and absorption coefficient, $n\left(\nu \right)\alpha \left(\nu \right)$, as a function of frequency (THz) for all studied glasses, where the power-law function was fit to our experimental data to determine the K and β parameters. Distinct trends dependent upon glass family are observed for the product of refractive index and absorption coefficient, following the same trends observed with individual THz refractive indices and absorption coefficients reported earlier. Higher THz optical properties are seen for tellurite glasses, followed by chalcogenide glasses, and then borosilicate glasses.

**Figure 6:**
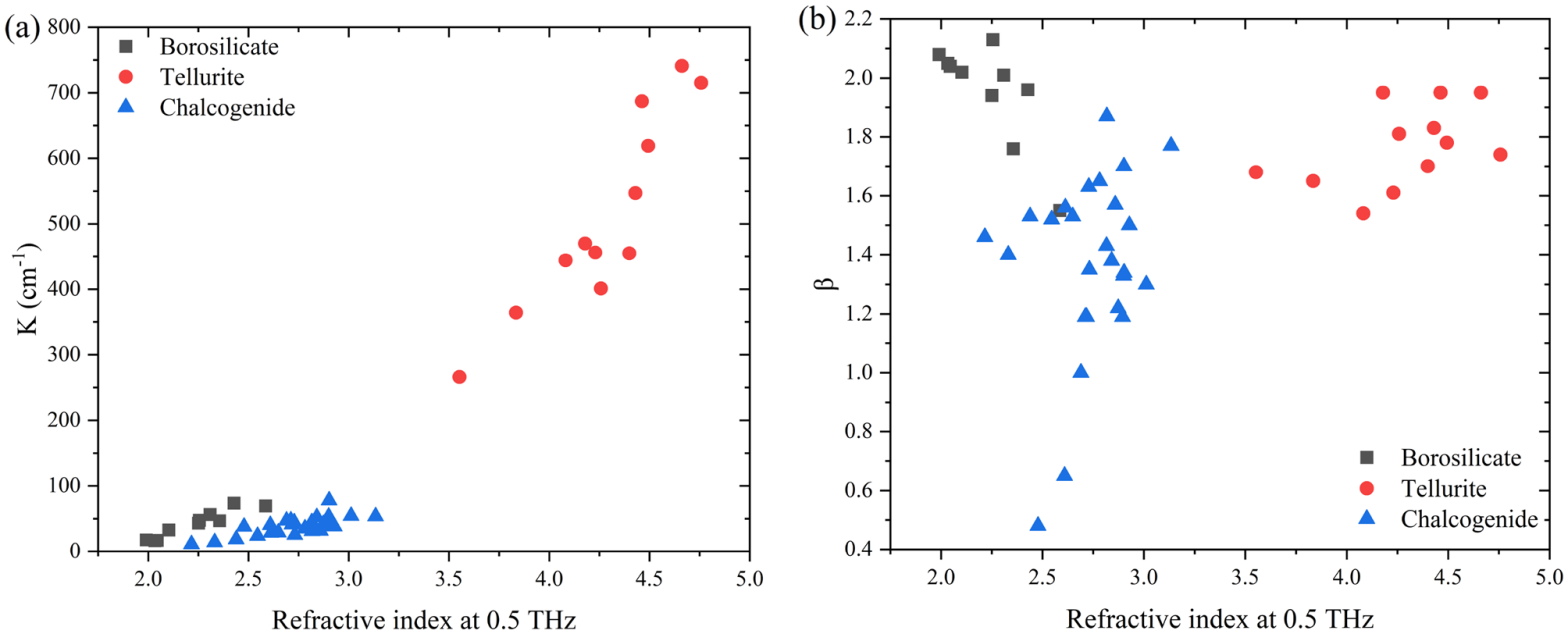
Relationship between (**a**) K and (**b**) β and 0.5 THz refractive index.

Figure 6 shows the (a) K and (b) β parameters for all studied glasses as a function of 0.5 THz refractive index. As seen with the THz refractive indices and absorption coefficients, distinct trends are observed for both power-law parameters. The K parameter is higher for glasses with higher polarizability and polarizable constituents, with tellurite glasses possessing the larges values, followed by borosilicate and chalcogenide glasses which share similar values. The borosilicate glasses of β parameters are ~ 2 in agreement with Naftaly et al.^14–16^. We observe distinct deviations in the case of chalcogenide glasses; however, our compositions are vastly different than those reported in literature and cover a wide, previously not reported, compositional space. The As_40_S_60_ and As_40_Se_60_ have the two highest β parameters within the chalcogenide family with 1.87 and 1.77, respectively, which is comparable to ~ 2 as reported by Storm et al.^19,20^. The remaining chalcogenide glasses record lower β parameters as low as 0.48 for vitreous selenium (Se). To the best of our knowledge, the β parameters for tellurite glasses have not been reported before. Tellurite glasses have β parameters between 1.61 and 1.95.

Our chalcogenide glasses cover vast compositional spaces within unary, binary, and ternary systems previously not reported in the literature with more variation in β parameters. However, in the case of borosilicate and tellurite glass systems, the compositional and chemistry variations result in more consistent β parameters comparable to those found in literature.

## Conclusion

Borosilicate, tellurite, and chalcogenide glass families were examined by THz-TDS determining THz optical properties, i.e., refractive indices and absorption coefficients, across the measured bandwidth. Our data reveals the ability to select appropriate glass family and composition to achieve desired THz properties for THz applications. Our K and β fitting parameters are comparable to the reported values in some cases, particularly for borosilicate and select chalcogenide glasses. Our results suggest that the β parameter can vary over a broad range of ~ 0.5–2, depending on the glass chemistry and nature of bonding, e.g., predominantly covalently bonded chalcogenide glasses.

## Data Availability

Data underlying the results presented in this paper are not publicly available at this time but may be obtained from the authors upon reasonable request.
